# Antimicrobial Activity of Chemokine CXCL10 for Dermal and Oral Microorganisms

**DOI:** 10.3390/antibiotics3040527

**Published:** 2014-10-23

**Authors:** Grant O. Holdren, David J. Rosenthal, Jianyi Yang, Amber M. Bates, Carol L. Fischer, Yang Zhang, Nicole K. Brogden, Kim A. Brogden

**Affiliations:** 1 Division of Pharmaceutics and Translational Therapeutics, Department of Pharmaceutical Sciences and Experimental Therapeutics, College of Pharmacy, The University of Iowa, Iowa City, IA 52242, USA; E-Mails: gholdren@gmail.com (G.O.H.); nicole-brogden@uiowa.edu (N.K.B.); 2 Dows Institute for Dental Research, College of Dentistry, The University of Iowa, Iowa City, IA 52242, USA; E-Mails: drosent@iastate.edu (D.J.R.); amber-bates@uiowa.edu (A.M.B.); carol-bratt@uiowa.edu (C.L.F.); 3 Department of Computational Medicine and Bioinformatics, The University of Michigan, 100 Washtenaw Avenue, Ann Arbor, MI 48109, USA; E-Mails: yangji@umich.edu (J.Y.); zhng@umich.edu (Y.Z.); 4 Periodontics, College of Dentistry, The University of Iowa, Iowa City, IA 52242, USA

**Keywords:** antimicrobial, CXCL10, IP-10, dermal microorganisms, oral microorganisms, SMAP28

## Abstract

CXCL10 (IP-10) is a small 10 kDa chemokine with antimicrobial activity. It is induced by IFN-γ, chemoattracts mononuclear cells, and promotes adhesion of T cells. Recently, we detected CXCL10 on the surface of the skin and in the oral cavity. In the current study, we used broth microdilution and radial diffusion assays to show that CXCL10 inhibits the growth of *Escherichia coli*, *Staphylococcus aureus*, *Corynebacterium jeikeium*, *Corynebacterium striatum*, and *Candida albicans* HMV4C, but not *Corynebacterium bovis*, *Streptococcus mutans*, *Streptococcus mitis*, *Streptococcus sanguinis*, *Fusobacterium nucleatum*, *Aggregatibacter actinomycetemcomitans*, *Poryphromonas gingivalis*, or *C. albicans* ATCC 64124. The reason for the selective antimicrobial activity is not yet known. However, antimicrobial activity of CXCL10 may be related to its composition and structure, as a cationic 98 amino acid residue molecule with 10 lysine residues, 7 arginine residues, a total net charge of +11, and a theoretical pI of 9.93. Modeling studies revealed that CXCL10 contains an α-helix at the N-terminal, three anti-parallel β-strands in the middle, and an α-helix at the C-terminal. Thus, CXCL10, when produced on the surface of the skin or in the oral cavity, likely has antimicrobial activity and may enhance innate antimicrobial and cellular responses to the presence of select commensal or opportunistic microorganisms.

## 1. Introduction

CXCL10 (IP-10) is a small 10 kDa chemokine. Structurally, it falls into the CXC family of chemokines, which differs from the C, CC, and CX3C families based on variations in the separation of N-terminal cysteines [1,2]. CXCL10 is induced by IFN-γ and produced by monocytes, fibroblasts, and endothelial cells [3], as well as activated neutrophils and eosinophils [4]. Under inflammatory situations, CXCL10 chemoattracts monocytes, macrophages, natural killer cells, dendritic cells, and cytotoxic and helper T cells (specifically Th1) [5,6] and promotes adhesion of T cells. CXCL10 is expressed in higher levels with multiple disease states, including cancer [5], infectious diseases [7], psoriasis [8], and various autoimmune diseases [9,10]. Along with the chemoattractant activity in immune responses, CXC chemokines are active in the regulation of angiogenesis. CXCL10 is missing the ELR (Glu-Leu-Arg) motif, indicating that it acts as an inhibitor of angiogenesis [11]. Additionally, members of the CXC chemokine family function as antimicrobial peptides, serving to enhance innate antimicrobial defense [2,12]. CXCL10 has antimicrobial activity against *Escherichia coli* [2,12], *Staphylococcus aureus* [2], *Listeria monocytogenes* [12], *Streptococcus pyogenes* [13], *Bacillus anthracis* [14,15], and *Candida albicans* [2].

Recently, we and others have detected CXCL10 in and on the surface of the skin [8,10] and in the oral cavity [16,17]. These findings suggest that CXCL10 may enhance the antimicrobial barriers in these areas. To test this, we assessed the antimicrobial activity of CXCL10 for microorganisms commonly found as commensals or pathogens on the skin and in the oral cavity.

## 2. Results

### 2.1. Activity of CXCL10 on Microorganisms Commonly Found on the Skin

CXCL10 had antimicrobial activity against select microorganisms that commonly inhabit the skin (Table 1 and Table 2). However, the activity of CXCL10 was also sensitive to the conditions of the antimicrobial assays, particularly the saline and media composition of the diluent used for each microorganism in the assay. SMAP28 was used as a control peptide and inhibited the growth of these microorganisms.

**Table 1:** Broth microdilution assays showing the minimal inhibitory concentration (MIC) and the minimal bactericidal concentration (MBC) of CXCL10 for microorganisms commonly found as commensals or pathogens on the skin.

Microorganism	CXCL10 µg/mL MIC (Standard Error)	CXCL10 µg/mL MBC (Standard Error)	SMAP28 µg/mL MIC (Standard Error)	SMAP28 µg/mL MBC (Standard Error)
*S. aureus*	>50.00	>50.00	4.17 (1.04)	6.25 (0.00)
*E. coli*	>50.00	>50.00	3.13 (0.00)	3.13 (0.00)
*C. bovis*	>50.00	>50.00	6.25 (0.00)	6.25 (0.00)
*C. striatum*	5.21 (1.04)	25.00 (0.00)	0.07 (0.01)	0.07 (0.01)
*C. jeikeium*	>50.00	>50.00	0.31 (0.00)	0.31 (0.00)

Minimal inhibitory concentration (MIC) and minimal bactericidal concentration (MBC) assays were performed in triplicate for each microorganism. *S. aureus* and *E. coli* were cultivated in Mueller Hinton Broth (MHB) at 37 °C for 24 h and *C. bovis*, *C. striatum*, and *C. jeikeium* were cultivated in Brain Heart Infusion Broth containing 0.1% Tween 80 at 37 °C (BHI-T80) for 48 h.

**Table 2:** Radial diffusion assays showing the minimal inhibitory concentration (MIC) of CXCL10 for microorganisms commonly found as commensals or pathogens on the skin.

Microorganism	CXCL10 µg/mL MIC (Standard Error)	SMAP28 µg/mL MIC (Standard Error)
*S. aureus*	125.89 (0.00)	10.49 (1.66)
*E. coli*	11.62 (4.23)	3.69 (0.75)
*C. bovis*	>200.00	17.24 (0.43)
*C. striatum*	3.42 (1.55)	8.00 (1.62)
*C. jeikeium*	21.52 (0.23)	4.44 (0.26)

Minimal inhibitory concentration (MIC) assays were performed in triplicate for each microorganism. *S. aureus* and *E. coli* were cultivated in Mueller Hinton Broth (MHB) at 37 °C for 24 h; and *C. bovis*, *C. striatum*, and *C. jeikeium* were cultivated in Brain Heart Infusion Broth containing 0.1% Tween 80 at 37 °C (BHI-T80) for 48 h.

*S. aureus* was resistant to CXCL10 in the broth microdilution assay (MIC, MBC > 50.00 μg/mL; Table 1) and the viable plate count assay (MIC > 100.00 μg/mL) [18], but susceptible to CXCL10 in the radial diffusion assay (MIC 125.89 μg/mL; Table 2). *E. coli* was resistant to CXCL10 in the broth microdilution assay (MIC, MBC > 50.00 μg/mL; Table 1), but susceptible to CXCL10 in viable plate count assay (MIC < 100.00 μg/mL) [19] and in the radial diffusion assay (MIC 3.13 μg/mL). *C. bovis* was resistant to CXCL10 in the broth microdilution assay (MIC, MBC > 50.00 μg/mL; Table 1); the viable plate count assay (MIC > 100.00 μg/mL) [20], and the radial diffusion assay (MIC > 200.00 μg/mL; Table 2). *C. jeikeium* was resistant to CXCL10 (MIC, MBC > 50.00 μg/mL) in the broth microdilution and radial diffusion assays. In contrast, *C. striatum* was sensitive to CXCL10 in the broth microdilution (MIC, 5.21 μg/mL; MBC 25.00 μg/mL) and radial diffusion (MIC, 19.59 μg/mL) assays.

### 2.2. Activity of CXCL10 on Microorganisms Commonly Found in the Oral Cavity

CXCL10 did not have antimicrobial activity against bacteria that commonly inhabit the oral cavity (Table 3). In radial diffusion assays, CXCL10 did not inhibit the growth *Streptococcus mutans*, *Streptococcus mitis*, *Streptococcus sanguinis*, *Fusobacterium nucleatum*, *Aggregatibacter actinomycetemcomitans*, or *Poryphromonas gingivalis*. Again, SMAP28 was used as a control peptide and inhibited the growth of these microorganisms.

**Table 3:** Radial diffusion assays showing the minimal inhibitory concentration (MIC) of CXCL10 for microorganisms commonly found as commensals or pathogens in the oral cavity.

Microorganism	CXCL10 µg/mL MIC (Standard Error)	SMAP28 µg/mL MIC (Standard Error)
*S. mutans*	>200.00	40.51 (2.70)
*S. mitis*	>200.00	125.89 (0.00)
*S. sanguinis*	>200.00	61.55 (2.80)
*F. nucleatum*	>1000.00	39.47 (5.62)
*P. gingivalis 381*	>1000.00	69.70 (22.74)
*P. gingivalis ATCC 33277*	>1000.00	74.42 (11.32)
*A. actinomycetemcomitans*	>1000.00	26.18 (3.81)

Assays were performed in triplicate for each microorganism. *S. mutans*, *S. mitis*, and *S. sanguinis* were cultivated in trypticase soy broth with yeast extract (TSBYE) at 37 °C; *F. nucleatum* was cultivated in Schaedler’s broth at 37 °C in an anaerobic environment; *P. gingivalis* was cultured in Tryptic Soy Broth supplemented with vitamin K1 and hemin at 37 °C in an anaerobic environment; and *A. actinomycetemcomitans* was cultivated in TSBYE at 37 °C in 5.0% CO_2_.

### 2.3. Activity of CXCL10 on C. albicans Commonly Found on the Skin and in the Oral Cavity

*C. albicans* ATCC 64124 was resistant to CXCL10 in the broth microdilution (MIC, MBC > 50.0 μg/mL) and radial diffusion (MIC > 50.0 μg/mL) assays (Table 4). In contrast, *C. albicans* HMV4C was resistant to CXCL10 in the broth microdilution assay (MIC, MBC > 50.0 μg/mL), yet susceptible to CXCL10 in the radial diffusion assay (MIC 23.90 μg/mL).

**Table 4:** Broth microdilution and radial diffusion assays showing the minimal inhibitory concentration (MIC) and the minimal bactericidal concentration (MBC) of CXCL10 for *C. albicans* commonly found as a commensal or pathogen on the skin and in the oral cavity.

Microorganism	CXCL10 µg/mL MIC (Standard Error)	CXCL10 µg/mL MBC (Standard Error)	SMAP28 µg/mL MIC (Standard Error)	SMAP28 µg/mL MBC (Standard Error)
*Broth microdilution assay*				
*C. albicans* ATCC 64124	>50.00	>50.00	12.50 (0.00)	12.50 (0.00)
*C. albicans* HMV4C	>50.00	>50.00	12.50 (0.00)	16.67 (4.17)
*Radial diffusion assay*				
*C. albicans* ATCC 64124	>1,000.00	n/a	39.73 (18.60)	n/a
*C. albicans* HMV4C	23.90 (10.38)	n/a	18.90 (2.13)	n/a

Minimal inhibitory concentration (MIC) assays were performed in triplicate for each strain. *C. albicans* ATCC 64124 and *C. albicans* HMV4C were grown on trypticase soy agar (TSA) overnight and then cultivated in RPMI 1640 at 37 °C for 3 h. n/a = not applicable.

### 2.4. Proposed Structure of CXCL10

**Figure 1:**
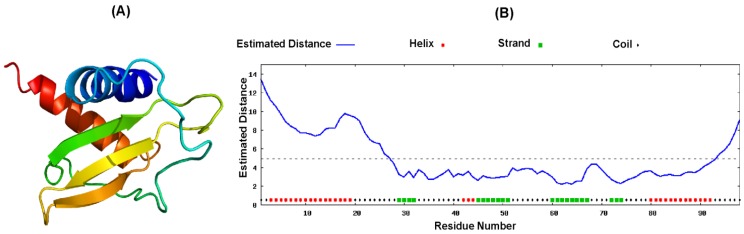
The structure modeling results of CXCL10. (**A**) I-TASSER model; (**B**) Estimated local accuracy.

The structure model of the protein CXCL10 was predicted by I-TASSER [21], and is shown in Figure 1A. The top templates used by I-TASSER include: 1o80A and 1lv9A. A confidence score (C-score) was used to estimate the accuracy of the I-TASSER model. This score is based on the clustering structural density/consensus significance of multiple threading templates. In the present study, the model was very accurate with a C-score of −0.47, estimated TM-score = 0.65 ± 0.13, and estimated RMSD = 4.8 ± 3.1 Å. Shown in Figure 1B is the local accuracy estimation. The residues between 29 and 93 have higher resolution (with predicted distance to native below 4 Å), which correspond to three anti-parallel β-strands in the middle and an α-helix at the C-terminal.

## 3. Discussion

In the current study, we found that CXCL10 has selective antimicrobial activity. In broth microdilution and radial diffusion assays, CXCL10 inhibits the growth of *E. coli*, *S. aureus*, *C. jeikeium*, *C. striatum*, and *C. albicans* HMV4C, but not *C. bovis*, *S. mutans*, *S. mitis*, *S. sanguinis*, *F. nucleatum*, *A. actinomycetemcomitans*, *P. gingivalis* strain 381 and ATCC 33277, or *C. albicans* ATCC 64124 (Table 1, Table 2, Table 3 and Table 4). Antimicrobial activity of CXCL10 may be related to its strong cationic composition and unique structure of three anti-parallel β-strands in the middle and an α-helix at the C-terminal. Thus CXCL10, when produced on the surface of the skin and in the oral cavity, may enhance innate antimicrobial and cellular responses to the presence of select microorganisms.

The skin and oral cavity are continually exposed to high concentrations of commensal and environmental microorganisms and both of these sites produce a vast diversity of antimicrobial substances as part of their innate immune host defense. This includes antimicrobial peptides, antimicrobial proteins, enzymes, and antimicrobial lipids. For example, the skin produces over 13 different antimicrobial substances [22]; the gingival crevicular fluid contains over 24 antimicrobial substances [23,24,25]; and saliva contains over 45 antimicrobial substances [23,24,25].

Cole and colleagues asked whether antimicrobial concentrations of CXCL10 could be achieved *in vivo* [12]. Based on the amount of CXCL10 produced from IFN-γ stimulated peripheral blood mononuclear cells and the density of mononuclear cells in lesions, they hypothesized that CXCL10 could be produced in areas of chronic inflammation in µg/mL amounts. Furthermore they propose that CXCL10 concentrations *in vivo* in plasma of individuals with meliodosis can also reach the MIC concentrations they observed in their study. We and others have recently detected CXCL10 on the surface of the skin and in the oral cavity. In a study of 23 subjects, surface skin wash fluid contained 0.0 to 59.3 pg/mL of CXCL10 (unpublished findings [26]); in a study of 20 subjects, saliva contained 634.0 ± 158.9 pg/mL CXCL10 [27]; in a study of 52 subjects, gingival crevicular fluid contained 36.0 to 3672.0 pg/mL CXCL10 [16]; and in a study of 6 subjects, gingival crevicular fluid contained 48.7 to 29,280.0 pg/mL CXCL10 [17]. These sites also have an incredible abundance and diversity of commensal and opportunistic microflora [28,29,30,31,32]. The presence of CXCL10 at these sites suggests that it may contribute to innate host defense.

CXCL10 has been reported to have antimicrobial activity against *E. coli* [2,12], *S. aureus* [2], *L. monocytogenes* [12], *S. pyogenes* [13], *B. anthracis* [14,15], and *C. albicans* [2]. In this study we extend these findings and report that CXCL10 has selective antimicrobial activity against microorganisms found on the skin and in the oral cavity, thus contributing to antimicrobial host defense by other antimicrobial peptides, antimicrobial proteins, enzymes, and antimicrobial lipids in these areas. Among microorganisms commonly found on the skin, *S. aureus* and *C. bovis* were more resistant to CXCL10 and *E. coli*, *C. jeikeium*, and *C. striatum* were more susceptible to CXCL10. Antimicrobial activity was likely dependent upon the saline and media composition of the diluents used in these assays. This was not an unexpected finding. Cole and colleagues nicely showed that the antimicrobial activity of CXCL10 for *E. coli* was dependent upon the saline concentration of the media [12]. *E. coli* was very susceptible to CXCL10 in 10 mM sodium phosphate buffer, pH 7.4 (MIC, 4.4 μg/mL); moderately susceptible in phosphate buffer with 50 mM NaCl (MIC, 25.0–50.0 μg/mL); and more resistant in phosphate buffer with 100 mM NaCl (MIC > 50.0 μg/mL). Similarly, the antimicrobial activity of CXCL10 for *S. pyogenes* was diminished in the presence of 150 mM NaCl [13].

The effect of saline and media composition of the diluent impacts the outcomes of the assays and it would be reasonable to assume that *in vivo*, antimicrobial activity of CXCL10 would be greater in microenvironments with higher concentrations of CXCL10 and lower amounts of saline and protein from serous fluid or plasma.

Yang and colleagues found that *S. aureus* was susceptible to CXCL10 [2]. In the current study, we found that *S. aureus* was resistant to CXCL10 in broth microdilution assays (MIC, MBC > 50.00 μg/mL; Table 1) yet susceptible to CXCL10 in radial diffusion assays (MIC 125.89 μg/mL; Table 2). The reason for this difference is not known but again may be related to the saline and media composition of the diluents used in the broth microdilution assay or may be related to differences among strains. Differences among strains do occur. For example, Yang and colleagues found that *C. albicans* was susceptible to CXCL10 [2]. We found that *C. albicans* ATCC 64124 was resistant to CXCL10 (MIC > 50.0 μg/mL in the broth microdilution assay; Table 4); *C. albicans* HMV4C was resistant to CXCL10 (MIC > 50.0 μg/mL in the broth microdilution assay; Table 4), yet *C. albicans* HMV4C was susceptible to CXCL10 (MIC 23.90 μg/mL in the radial diffusion assay; Table 4).

Interestingly, bacteria commonly found in the oral cavity were resistant to CXCL10. *C. albicans*, an opportunistic pathogen on the skin and in the oral cavity, was resistant or susceptible to CXCL10, depending upon the strain. Differences among strains do occur and this would warrant using a number of strains of each species in subsequent work.

The exact mechanism for the antimicrobial activity of CXCL10 for bacteria is not yet known but is likely related to its unique composition and structure. CXCL10 contains 10 lysine residues, 7 arginine residues, has a net charge of +11, and has a theoretical pI of 9.93. This likely facilitates the physical attraction of CXCL10 for the negatively charged microbial surface [12] and activity is influenced by the composition of components in the surrounding environment. Antimicrobial activity of CXCL10 is known to decrease in complex microbiological media and antimicrobial activity rapidly decreases as the concentration of NaCl increases in assay diluents [12,13]. What happens next is not well known. CXCL10 may possibly interact and form pores in the microbial cytoplasmic membrane. Alternately, CXCL10 may target specific cytoplasmic membrane proteins. One proposed target is FtsX, an integral cytoplasmic inner membrane protein thought to form the substrate translocation channel of a putative ABC transporter [14]. In cells treated with CXCL10 and processed for immunoelectron microscopy, CXCL10 bound to the bacterial cell membrane and was associated with loss of cellular integrity [14].

**Figure 2:**
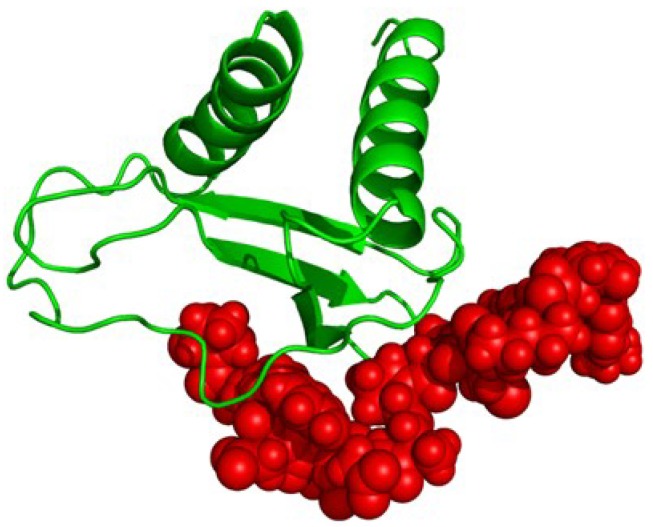
The CXCL10 (in green cartoon) is predicted to interact with a peptide (in red spheres).

We wanted to compare the modeled structure of CXCL10 with the structure of CXCL9. Egesten and colleagues assessed the structure of chemokine CXCL9 (MIG) with antimicrobial activity [13]. The N-terminus of CXCL9 contained a region composed of 3 antiparallel β-strands and the C-terminus contained a region composed of an α-helix. When areas of this molecule were synthesized and tested against *S. pyogenes*, the N-terminus did not have antimicrobial activity but the C-terminus did [13]. Yang and colleagues assessed the structure of CXCL10 [2]. CXCL10 had a secondary structure with a number of uniquely rich segments. It is rich in positively charged amino acid residues and has a uniquely positive charged C-terminus. In the current study, we found that the global topology of CXCL10 was similar to that of CXCL9 (Figure 1A): the middle of CXCL10 contained a region composed of 3 antiparallel β-strands and the C-terminus contained a region composed of an α-helix (Figures 1A). The major difference is that our model contains one α-helix at the N-terminus (Figure 1A). When further modeled, it would appear that the C-terminus α-helix would be expected to bind to other proteins and thus may be involved in antimicrobial activity similar to that proposed for CXCL9 (Figure 2).

## 4. Experimental Section

### 4.1. Microorganisms and Culture Conditions

Representative microorganisms commonly found on the skin surface were used and cultivated as previously described [33]. *S. aureus* ATCC 29213 was cultivated in Mueller Hinton Broth (MHB) at 37 °C for 24 h; *E. coli* ATCC 12795 was cultivated in MHB at 37 °C for 24 h; and *C. bovis* ATCC 7715, *C. jeikeium* ATCC 43734, and *C. striatum* ATCC 7094 were cultivated in Brain Heart Infusion Broth containing 0.1% Tween 80 (BHIB-T80) at 37 °C for 48 h.

Representative microorganisms commonly found in the oral cavity were also used and cultivated as previously described [33,34]. *S. mutans* ATCC 25175, *S. mitis* ATCC 49456, and *S. sanguinis* ATCC 10556 were cultivated in trypticase soy broth with 0.6% yeast extract (TSBYE) at 37 °C; *F. nucleatum* ATCC 25586 was cultivated in Schaedler’s broth at 37 °C in an anaerobic environment; *P. gingivalis* strain 381 and *P. gingivalis* ATCC 33277 were cultured in Tryptic Soy Broth (TSB; Difco Laboratories, Detroit, MI, USA) supplemented with vitamin K1 and hemin (Sigma Chemical Co., St Louis, MO, USA) at 37 °C in an anaerobic environment; and *A. actinomycetemcomitans* ATCC 43718 was cultivated in TSBYE at 37 °C in 5.0% CO_2_.

*C. albicans* ATCC 64124 and *C. albicans* HMV4C were grown on trypticase soy agar (TSA) and cultivated in RPMI 1640 at 37 °C for 3 h. Their identities were confirmed with a Bruker Daltonik MALDI Biotyper (State Hygienic Laboratory, University of Iowa Research Park, Coralville, IA, USA).

Prior to the antimicrobial assays, all cultures were transferred to fresh medium and incubated for 3 h in their respective culture conditions. Cultures were then adjusted to contain ~1.0 × 10^8^ cfu/mL (optical density 0.108; 600 nm; Spectronic 20D1, Thermo Fisher Scientific, Inc., Waltham, MA, USA) and then diluted to 10^−2^ to 10^−3^-fold in fresh media (depending upon the specific microorganism).

### 4.2. Chemokine and Antimicrobial Peptide

Recombinant CXCL10 (Peprotech, Rocky Hill, NJ, USA) was used. CXCL10 was suspended in 0.01% acetic acid to a concentration of 1000.0 μg/mL, gently mixed, dispensed as 100 μL aliquots into cryotubes, and frozen until use.

Sheep myeloid antimicrobial peptide (SMAP) 28 (NeoMPS, Inc., San Diego, CA, USA) was used as a positive control peptide [35] with antimicrobial activity against many of the microorganisms used in this study [36]. SAMP28 is robust and antimicrobial activity is not affected *in vitro* [37] or *in vivo* [38] by the presence of complex microbiological media and increased concentrations of NaCl. SMAP28 was suspended in 0.01% acetic acid to a concentration of 1000.0 µg/mL, gently mixed, dispensed as 100 μL aliquots into cryotubes, and frozen until use.

### 4.3. Broth Microdilution Assay

A broth microdilution assay was used to determine the antimicrobial activities of CXCL10 and SMAP28 for bacteria [35] and a modified broth microdilution assay was used to determine the antimicrobial activities of CXCL10 and SMAP28 for *C. albicans* [39]. Briefly, 0.01% acetic acid was added to the wells of column 1 to 12 of microtiter plates (Immulon 1 plates, ISC Bioexpress, Kaysville, UT, USA). 100 μL of CXCL10 or SMAP28 was then added to the wells in column 1 and diluted 2-fold from column 1 to column 10 using a multi-channel pipetter. 100 μL of bacterial culture was added to each well from column 1 to column 11. Column 11 served as the positive growth control (in diluent). 100 μL of sterile culture media was added to the wells in column 12 and used as the sterility controls and plate blanks. Alternately, 100 µL of a 3 h *C. albicans* culture in RPMI with resazurin (Alamar Blue, Invitrogen Corp., Carlsbad, CA, USA) was added to each well from column 1 to column 11. Column 11 served as the positive growth control (in diluent). 100 μL of sterile RPMI with resazurin was added to the wells in column 12 and used as the sterility controls and plate blanks. After set up, the plates were incubated overnight at 37 °C in the appropriate culture conditions. The microbial growth in the presence of CXCL10 or SMAP28 was assessed at 600 nm in the spectrophotometer (PowerWaveX, Bio-Tek instruments, Inc., Winooski, VT, USA). *C. albicans* growth in the presence of CXCL10 or SMAP28 was assessed by measuring the metabolic reduction of resazurin to resorufin using an excitation wavelength of 544 nm and an emission wavelength of 590 nm (SpectraMax M2e Multi-Mode Microplate Reader, Molecular Devices, LLC, Sunnyvale, CA, USA).

The minimal inhibitory concentration (MIC) was determined to be the lowest concentration of peptides that inhibited microbial growth by more than 50% of the positive growth control in each row. The wells were cultured onto the respective agars and the minimal bactericidal concentration (MBC) was determined to be the lowest concentration of peptides that inhibited microbial growth on agar. Broth microdilution assays were performed in triplicate for each microorganism.

### 4.4. Radial Diffusion Assay

A radial diffusion assay was used to determine the antimicrobial activities of CXCL10 and SMAP28 for *S. aureus*, *E. coli*, *C. bovis*, *C. jeikeium*, and *C. striatum* [12] and a modified radial diffusion assay was used to determine the antimicrobial activities of CXCL10 and SMAP28 for *S. mutans*, *S. mitis*, *S. sanguinis*, *F. nucleatum*, *A. actinomycetemcomitans*, and *P. gingivalis* [36]. Briefly, 4.0 × 10^6^ microorganisms were suspended in 25.0 mL of 1% agarose in 0.01 sodium phosphate buffer, pH 7.4 at 50 °C and pipetted into square Petri dishes. When solidified, 2.0 mm diameter wells were punched into the agar. Five microliters of CXCL10 or SMAP28 at 1000.0, 500.0, 250.0, 125.0, 62.5, and 0.0 μg/mL were added per well. The plates were incubated at 37 °C. After 3 h, 10.0 mL of 1.0% agarose containing 6.0% of the respective microbial media was added and allowed to solidify. The plates were incubated at 37 °C in their respective culture conditions. At 18 h, clear zones representing microbial growth inhibition were measured. Radial diffusion units were calculated as the diameter of the clear zone less the 2.0 mm diameter of the well ×10. The radial diffusion units were plotted against the log_10_ of the peptide concentration and the MIC was determined to be anti-log_10_ of the *x*-intercept. The radial diffusion assays were performed in triplicate for each microorganism.

### 4.5. Viable Plate Count Assay

A viable plate count assay was used to assess the direct effects of CXCL10 on the viability of dermal and oral microorganisms. For this, microorganisms were cultivated as described above, adjusted to contain ~1.0 × 10^8^ cfu/mL (optical density 0.108; 600 nm; Spectronic 20D1, Thermo Fisher Scientific, Inc., Waltham, MA, USA), and then diluted 10^−4^-fold in sterile water. 50 μL containing ~500 cfu, was added to 50 μL of CXCL10 in 0.01% acetic acid, SMAP28 in 0.01% acetic acid, or 0.01% acetic acid. After incubation for one hour at 37 °C, 25 μL was removed and spotted in triplicate on the respective agar for each microorganism. After incubation for 24 h, colonies were counted.

### 4.6. Modeling the Structure of CXCL10

The structure of CXCL10 was predicted using the iterative threading assembly refinement (I-TASSER) server [21,40]. Briefly, the 98 amino acid residue FASTA format sequence of CXCL10 was threaded against the PDB library using the meta-threading algorithm LOMETS to identify homologues templates. Fragments that were excised from the threading templates were then reassembled into full-length models by replica exchange Monte Carlo simulations with the threading unaligned regions (mainly loops) built by ab initio folding. The lowest free-energy conformation was selected by clustering the Monte Carlo simulation structures using SPICKER39. Next, fragment assembly simulation was performed again starting from the SPICKER cluster centroids, where the spatial restraints collected from both the LOMETS templates and the analogy PDB structures by TM-align were used to guide the reassembly simulations. Finally, the models were refined in the atomic-level by the fragment guided molecular dynamics (FG-MD) simulations.

To examine the biological function of the CXCL10 sequence, the COACH algorithm [41] was used to match the I-TASSER model to the protein function library BioLiP [42]. As shown in Figure 2, the CXCL10 was predicted to interact with a peptide (18 residues from the extracellular portion of the receptor CXCR-1) using the template interleukin-8 (PDB ID: 1ilpA).

## 5. Conclusions

CXCL10, when produced on the surface of the skin and in the oral cavity, may have a broader role than previously thought in enhancing innate antimicrobial and cellular responses to the presence of microorganisms. In this study we show that CXCL10 has antimicrobial activity against select microorganisms commonly found on the skin or in the oral cavity. Antimicrobial activity may be due to the unique characteristics of CXCL10.
